# Clinicopathological characteristics and survival outcomes in spindle cell carcinoma (SpCC) of the breast: A SEER population-based study

**DOI:** 10.1097/MD.0000000000044851

**Published:** 2025-10-03

**Authors:** Yushi Sun, Heyan Chen, Ke Wang, Yang Liu

**Affiliations:** a Department of Endocrinology, The First Affiliated Hospital of Xi’an Jiaotong University, Xi’an, Shaanxi, China; b Department of Breast Surgery, The First Affiliated Hospital of Xi’an Jiaotong University, Xi’an, Shaanxi, China.

**Keywords:** clinicopathological characteristics, invasive ductal carcinoma, prognosis, SEER, spindle cell carcinoma

## Abstract

Spindle cell carcinoma (SpCC) of the breast is a rare entity. The aim of this study was to provide more information for understanding this disease and to improve the management of it in the clinic. Patients with SpCC and invasive ductal carcinoma (IDC) of the breast were identified through the surveillance, epidemiology, and end results (SEER) database (2001–2018). 227 patients with SpCC and 565,388 patients with breast IDC were enrolled in the present cohort study. Comparative analyses were performed to investigate the heterogeneity in the clinicopathological characteristics and survival outcomes between these 2 groups. Propensity score matching (PSM) was used to balance the influences of baseline clinicopathological differences. The multivariate Cox proportional hazard model was carried out to identify potential prognostic factors of SpCC. Compared with IDC, patients with SpCC had a higher proportion of older patients and white individuals, a higher tumor grade, a lower tumor stage, a larger tumor size, a higher incidence of distant metastasis, a lower rate of lymph node involvement, a higher proportion of triple-negative breast cancer (TNBC) and less access to therapeutics. The prognosis of SpCC was profoundly poorer than that of IDC, whether before or after PSM. Subgroup analysis further showed that SpCC-TNBC had a worse clinical outcome than IDC-TNBC. Finally, we found that older age, advanced T stage, N stage and M stage were all risk factors for SpCC. SpCC of the breast presented with increasing aggressive behavior in comparison with IDC and inferior clinical outcome than IDC for both the whole group and the TNBC subgroup. Distinguishing SpCC from IDC is critical for improving treatment efficacy; therefore, further research must focus on this rare but aggressive disease.

## 1. Introduction

Spindle cell carcinoma (SpCC) of the breast is a rare entity, which only comprises 0.02 to 0.3% of all invasive breast cancers.^[1,2]^ SpCC of the breast is the most common subtype of metaplastic breast carcinoma^[3]^ and is characterized by the infiltration of monophasic spindle cell population devoid of any morphologic epithelial component and mainly positive for p63, CD10, and CK14.^[1,3–5]^ SpCC is characterized as a variant of metaplastic mammary carcinoma, and other subtypes are including adenosquamous carcinoma, fibromatosis-like metaplastic carcinoma, squamous cell carcinoma, metaplastic breast cancer with mesenchymal differentiation.^[3,6]^

SpCC of the breast usually has a triple-negative phenotype (ER, PR and HER2 are negative).^[7]^ Previous studies have assessed the clinical presentations of SpCC of the breast.^[1–5,7,8]^ Unfortunately, most of the studies were case reports or small single-center retrospective analyses. Of note, patients with SpCC are given the treatment options empirically as for invasive ductal carcinoma (IDC).^[1,8]^ Currently, treatment strategies for SpCC vary considerably, particularly in the choice of surgical approach and the use of radiotherapy.^[9,10]^ However, the clinical features and prognostic profiles of SpCC are poorly understood so far. What’s more, accurate information concerning the comparison of SpCC and IDC is still lacking. Thus, there is an urgent need to provide more information for a better understanding of the clinicopathological characteristics in the SpCC population and evaluate potential prognostic differences between SpCC and IDC.

In this study, we performed comparisons of the clinicopathological characteristics and prognosis between SpCC and IDC of the breast for the first time. We further compared the clinicopathologic characteristics and prognosis between the IDC-TNBC and SpCC-TNBC subgroups before or after propensity score matching (PSM). The aim of this study was to provide an informative reference for the treatment of SpCC of the breast in the clinic.

## 2. Materials and methods

### 2.1. Patient selection

Data from our study were collected from the surveillance, epidemiology, and end results (SEER) database, which is one of the world’s largest open cancer databases, established by the National Cancer Institute and updated in November 2018.^[11,12]^ Since the SEER database is available to users worldwide, informed consent of patients is not required for our study. This study was exempt from the approval process of the ethics committee of the First Affiliated Hospital of Xi’an Jiaotong University.

### 2.2. Patient and variable selection

**Figure 1.**
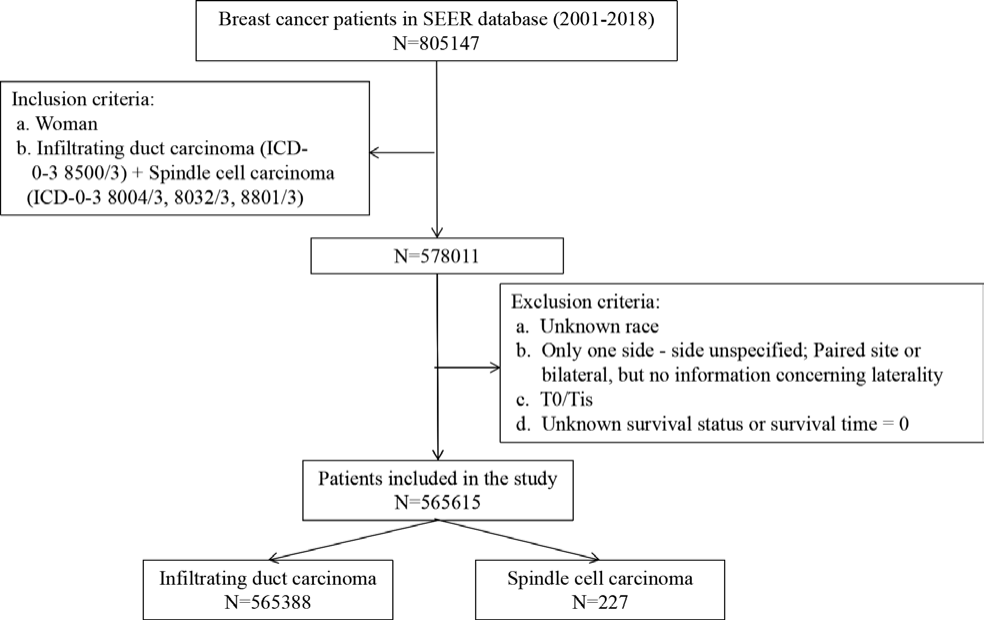
Flowchart of patients identified in this study. SEER = surveillance epidemiology and end results.

Patients meeting the following criteria were included: Female patients (2001–2018); Pathologically diagnosed as infiltrating duct carcinoma (IDC) (ICD-0-3 8500/3) and pure SpCC (ICD-0-3 8004/3, 8032/3, 8801/3). Then, patients meeting the following criteria were excluded: patients with the follow-up type of autopsy/death certificate only; patients with unknown race or unknown/indefinite site of laterality; and patients with T0 or Tis. In total, 565,615 patients were included in this study (including IDC = 565,388 and SpCC = 227), and the flow chart of patient screening is shown in Figure 1.

Clinicopathological characteristics included age at diagnosis, race, marital status, laterality, grade, AJCC stage (BREAST – ADJUSTED AJCC 6TH STAGE), tumor size, node involvement, metastasis sites, ER status, PR status, HER2 status, molecular subtype, chemotherapy, radiation, surgery, survival in months and causes of death from the SEER database. Age was divided into <40 years old, 40 to 60 years old and >60 years old. Patients were categorized into 4 subtypes: HR + HER2−, HR + HER2+, HR-HER2+, and HR-HER2− (triple negative). Patients were stratified by breast cancer histologic grade: Grade I (well differentiated), Grade II (moderately differentiated), Grade III (poorly differentiated), Grade IV (undifferentiated), and Unknown (unknown histologic grade).

### 2.3. Outcomes

The primary endpoint of our study was overall survival (OS). OS was defined as the time from diagnosis to death from any reason. The second endpoint of the study was breast cancer-specific survival (BCSS). BCSS was defined as the time between initial diagnosis and death from breast cancer.

### 2.4. Statistical analysis

Clinicopathological features were compared between the IDC group and the SpCC group using Pearson chi-square test or Fisher exact test. Kaplan–Meier curves and log-rank tests were conducted to analyze the OS and BCSS between IDC and SpCC patients. In addition, we used multivariate Cox proportional hazard models to identify other variables that may affect the prognosis of SpCC patients. PSM is a reliable tool that can decrease selection bias and balance covariates across treatment groups in non-randomized studies.^[13]^ In this study, PSM was performed to balance the differences in baseline characteristics (age at diagnosis, race, marital status, laterality, grade, TNM stage, tumor size, lymph node involvement, metastasis stage, ER status, PR status, HER2 status, molecular subtype, chemotherapy, radiation, surgery) between groups at a 1:5 ratio using the “MatchIt” R package and the nearest neighbor matching with caliper 0.05. A two-sided *P* value <.05 was considered statistically significant. All statistical analyses were performed using R software v4.1.1 (http://www.r-project.org).

## 3. Results

### 3.1. Clinicopathological characteristics

A total of 227 patients with SpCC of the breast and 565,388 with IDC of the breast were eligible for inclusion in this study. The differences in the baseline characteristics between the 2 cohort groups are presented in Table 1. Compared with IDC, patients with SpCC had a higher proportion of patients older than 60 years of age (55.1% vs 45.9%, *P* = .014), a higher proportion of white individuals (84.6% vs 79.1%, *P* = .043), a higher tumor grade (III–IV, 45.4% vs 36.1%, *P* < .001), a lower tumor stage (III–IV, 12.3% vs 15.7%, *P* < .001), a larger tumor size (>5 cm, 18.9% vs 8.1%, *P* < .001), a higher incidence of distant metastasis (M1, 5.7% vs 4.3%, *P* < .001), a lower rate of lymph node involvement (N1–N3, 8.4% vs 33.1%, *P* < .001), and a higher proportion of triple-negative breast cancer (TNBC) (26.4% vs 6.5%, *P* < .001) (Table 1).

**Table 1:** Baseline characteristics of patients with spindle cell carcinoma (SpCC) and invasive ductal carcinoma (IDC) before or after PSM.

Characteristics	Before PSM			After PSM		
	IDC	SpCC	*P*-value	IDC	SpCC	*P*-value
All patients	N = 565,388	N = 227		N = 1135	N = 227	
Age at diagnosis (%)			.014			.549
<40	37,601 (6.7)	16 (7.0)		63 (5.6)	16 (7.0)	
40–60	268,140 (47.4)	86 (37.9)		463 (40.8)	86 (37.9)	
>60	259,647 (45.9)	125 (55.1)		609 (53.7)	125 (55.1)	
Race (%)			.043			.659
Black	64,004 (11.3)	24 (10.6)		100 (8.8)	24 (10.6)	
White	447,253 (79.1)	192 (84.6)		985 (86.8)	192 (84.6)	
Other	54,131 (9.6)	11 (4.8)		50 (4.4)	11 (4.8)	
Marital status (%)			.142			.949
Married	315,200 (55.7)	116 (51.1)		578 (50.9)	116 (51.1)	
Single	223,742 (39.6)	95 (41.9)		470 (41.4)	95 (41.9)	
Unknown	26,446 (4.7)	16 (7.0)		87 (7.7)	16 (7.0)	
Laterality (%)			.867			.808
Left	287,020 (50.8)	117 (51.5)		598 (52.7)	117 (51.5)	
Right	278,368 (49.2)	110 (48.5)		537 (47.3)	110 (48.5)	
Grade (%)			<.001			.707
I–II	306,134 (54.1)	34 (15.0)		187 (16.5)	34 (15.0)	
III–IV	204,105 (36.1)	103 (45.4)		483 (42.6)	103 (45.4)	
Unknown	55,149 (9.8)	90 (39.6)		465 (41.0)	90 (39.6)	
Stage (%)			<.001			.975
I–II	456,191 (80.7)	136 (59.9)		689 (60.7)	136 (59.9)	
III–IV	88,705 (15.7)	28 (12.3)		137 (12.1)	28 (12.3)	
Unknown	20,492 (3.6)	63 (27.8)		309 (27.2)	63 (27.8)	
T stage (%)			<.001			.971
T1–T2	487,688 (86.3)	115 (50.7)		570 (50.2)	115 (50.7)	
T3–T4	45,758 (8.1)	43 (18.9)		211 (18.6)	43 (18.9)	
Unknown	31,942 (5.6)	69 (30.4)		354 (31.2)	69 (30.4)	
N stage (%)			<.001			.997
N0	357,886 (63.3)	146 (64.3)		727 (64.1)	146 (64.3)	
N1–N3	187,076 (33.1)	19 (8.4)		95 (8.4)	19 (8.4)	
Unknown	20,426 (3.6)	62 (27.3)		313 (27.6)	62 (27.3)	
M stage (%)			<.001			.966
M0	535,287 (94.7)	162 (71.4)		815 (71.8)	162 (71.4)	
M1	24,223 (4.3)	13 (5.7)		68 (6.0)	13 (5.7)	
Unknown	5878 (1.0)	52 (22.9)		252 (22.2)	52 (22.9)	
ER status (%)						
Negative	116,673 (20.6)	125 (55.1)		657 (57.9)	125 (55.1)	
Positive	417,731 (73.9)	17 (7.5)	<.001	79 (7.0)	17 (7.5)	.735
Borderline/Unknown	30,984 (5.5)	85 (37.4)		399 (35.2)	85 (37.4)	
PR status (%)						
Negative	171,435 (30.3)	125 (55.1)		657 (57.9)	125 (55.1)	
Positive	358,495 (63.4)	16 (7.0)	<.001	70 (6.2)	16 (7.0)	.708
Borderline/Unknown	35,458 (6.3)	86 (37.9)		408 (35.9)	86 (37.9)	
HER2 status (%)						
Negative	237,340 (42.0)	75 (33.0)		393 (34.6)	75 (33.0)	
Positive	53,009 (9.4)	(0.4)	<.001	5 (0.4)	1 (0.4)	.900
Borderline/Unknown	275,039 (48.6)	151 (66.5)		737 (64.9)	151 (66.5)	
Subtypes (%)			<.001			.408
HR-/HER2-	36,946 (6.5)	60 (26.4)		349 (30.7)	60 (26.4)	
HR-/HER2+	16,091 (2.8)	0 (0.0)				
HR+/HER2−	200,016 (35.4)	13 (5.7)		44 (3.9)	13 (5.7)	
HR+/HER2+	36,758 (6.5)	1 (0.4)		5 (0.4)	1 (0.4)	
Unknown	275,577 (48.7)	153 (67.4)		737 (64.9)	153 (67.4)	
Surgery (%)			.076			.902
No/Unknown	39,143 (6.9)	23 (10.1)		109 (9.6)	23 (10.1)	
Yes	526,245 (93.1)	204 (89.9)		1026 (90.4)	204 (89.9)	
Radiation (%)			<.001			.879
No	272,738 (48.2)	146 (64.3)		739 (65.1)	146 (64.3)	
Yes	292,650 (51.8)	81 (35.7)		396 (34.9)	81 (35.7)	
Chemotherapy (%)			.009			.332
No/Unknown	313,884 (55.5)	146 (64.3)		688 (60.6)	146 (64.3)	
Yes	251,504 (44.5)	81 (35.7)		447 (39.4)	81 (35.7)	

Borderline: The test result falls within a borderline range and cannot be definitively classified as positive or negative. Unknown: The receptor status is unknown, possibly due to undetected, test failure, or the result not being recorded.

IDC = invasive ductal carcinoma, PSM = Propensity score matching, SpCC = spindle cell carcinoma.

**Figure 2.**
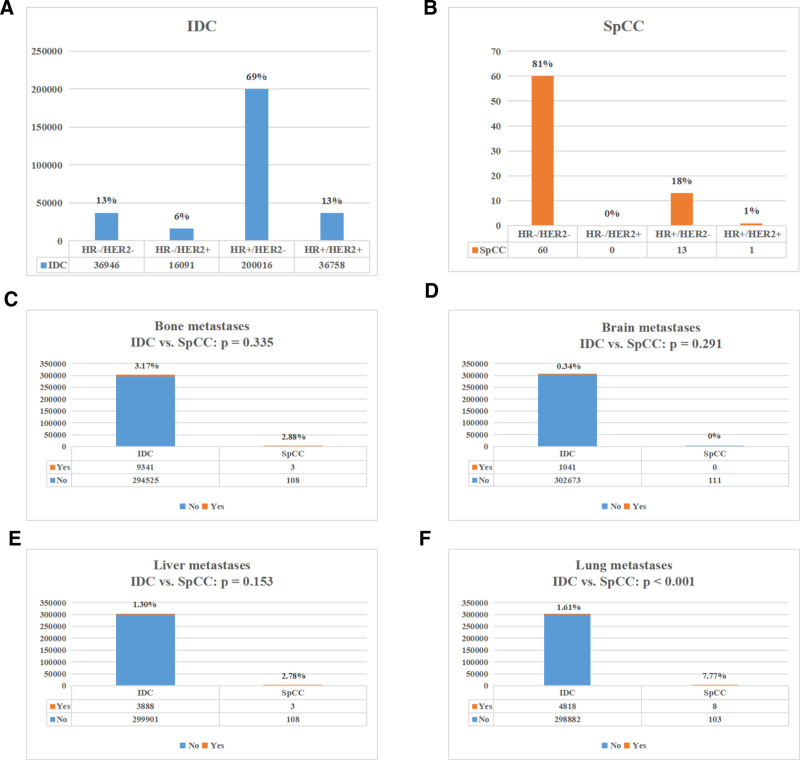
Clinicopathologic characteristics of study cohort. (A) Distribution of molecular subtype in IDC group. (B) Distribution of molecular subtype in SpCC group. (C–F) Comparisons of the proportion of different distant metastases (bone metastases; brain metastases; liver metastases; lung metastases) in IDC and SpCC group. IDC = invasive ductal carcinoma, SpCC = spindle cell carcinoma.

Concerning treatment options, patients with SpCC were less likely to receive radiotherapy (35.7% vs 51.8%, *P* < .001) and chemotherapy (35.7% vs 44.5%, *P* = .009) than IDC patients, while no significant difference was detected in the surgical management rate between the 2 groups. In comparison with IDC, patients with SpCC exhibited a higher rate of lung metastasis (7.77% vs 1.61%, *P* < .001) (Fig. 2).

Since TNBC was a molecular subtype of breast cancer with poor prognosis,^[14]^ and TNBC accounted for the largest proportion of SpCC patients with a clear molecular type, we further compared the clinicopathologic characteristics between the IDC-TNBC and SpCC-TNBC subgroups. Compared with IDC-TNBC, patients with SpCC-TNBC had a higher proportion of older patients and white individuals, a lower tumor grade, a lower rate of lymph node involvement, and less access to chemotherapy (Table 2).

**Table 2:** Comparisons the clinicopathologic characteristics between the IDC-TNBC and SpCC-TNBC subgroups before or after PSM.

Characteristics	Before PSM			After PSM		
	IDC-TNBC	SpCC-TNBC	*P*-value	IDC-TNBC	SpCC-TNBC	*P*-value
All patients	N = 36,946	N = 60		N = 300	N = 60	
Age at diagnosis (%)			<.001			.933
<40	3800 (10.3)	3 (5.0)		12 (4.0)	3 (5.0)	
40–60	18,030 (48.8)	15 (25.0)		78 (26.0)	15 (25.0)	
>60	15,116 (40.9)	42 (70.0)		210 (70.0)	42 (70.0)	
Race (%)			.019			.588
Black	7963 (21.6)	7 (11.7)		23 (7.7)	7 (11.7)	
White	26,020 (70.4)	52 (86.7)		271 (90.3)	52 (86.7)	
Other	2963 (8.0)	1 (1.7)		6 (2.0)	1 (1.7)	
Marital status (%)			.51			.85
Married	19,653 (53.2)	29 (48.3)		156 (52.0)	29 (48.3)	
Single	15,355 (41.6)	29 (48.3)		133 (44.3)	29 (48.3)	
Unknown	1938 (5.2)	2 (3.3)		11 (3.7)	2 (3.3)	
Laterality (%)			.748			1
Left	18,931 (51.2)	29 (48.3)		145 (48.3)	29 (48.3)	
Right	18,015 (48.8)	31 (51.7)		155 (51.7)	31 (51.7)	
Grade (%)			<.001			.826
I–II	5352 (14.5)	10 (16.7)		60 (20.0)	10 (16.7)	
III–IV	26,604 (72.0)	25 (41.7)		117 (39.0)	25 (41.7)	
Unknown	4990 (13.5)	25 (41.7)		123 (41.0)	25 (41.7)	
Stage (%)			.083			.92
I–II	28,737 (77.8)	51 (85.0)		255 (85.0)	51 (85.0)	
III-IV	7317 (19.8)	6 (10.0)		33 (11.0)	6 (10.0)	
Unknown	892 (2.4)	3 (5.0)		12 (4.0)	3 (5.0)	
T stage (%)			.128			.931
T1–T2	30,595 (82.8)	44 (73.3)		220 (73.3)	44 (73.3)	
T3–T4	5423 (14.7)	13 (21.7)		68 (22.7)	13 (21.7)	
Unknown	928 (2.5)	3 (5.0)		12 (4.0)	3 (5.0)	
N stage (%)			<.001			.915
N0	23,009 (62.3)	53 (88.3)		263 (87.7)	53 (88.3)	
N1–N3	13,344 (36.1)	5 (8.3)		29 (9.7)	5 (8.3)	
Unknown	593 (1.6)	2 (3.3)		8 (2.7)	2 (3.3)	
M stage (%)			.144			.427
M0	34,917 (94.5)	58 (96.7)		292 (97.3)	58 (96.7)	
M1	1884 (5.1)	1 (1.7)		1 (0.3)	1 (1.7)	
Unknown	145 (0.4)	1 (1.7)		7 (2.3)	1 (1.7)	
Surgery (%)			1			1
No/Unknown	3339 (9.0)	5 (8.3)		22 (7.3)	5 (8.3)	
Yes	33,607 (91.0)	55 (91.7)		278 (92.7)	55 (91.7)	
Radiation (%)			.127			.924
No	18,219 (49.3)	36 (60.0)		175 (58.3)	36 (60.0)	
Yes	18,727 (50.7)	24 (40.0)		125 (41.7)	24 (40.0)	
Chemotherapy (%)			<.001			.741
No/Unknown	8123 (22.0)	32 (53.3)		150 (50.0)	32 (53.3)	
Yes	28,823 (78.0)	28 (46.7)		150 (50.0)	28 (46.7)	

IDC = invasive ductal carcinoma, PSM = Propensity score matching, SpCC = spindle cell carcinoma, TNBC = triple-negative breast cancer.

### 3.2. Comparison of survival between SpCC and IDC of the breast

**Figure 3.**
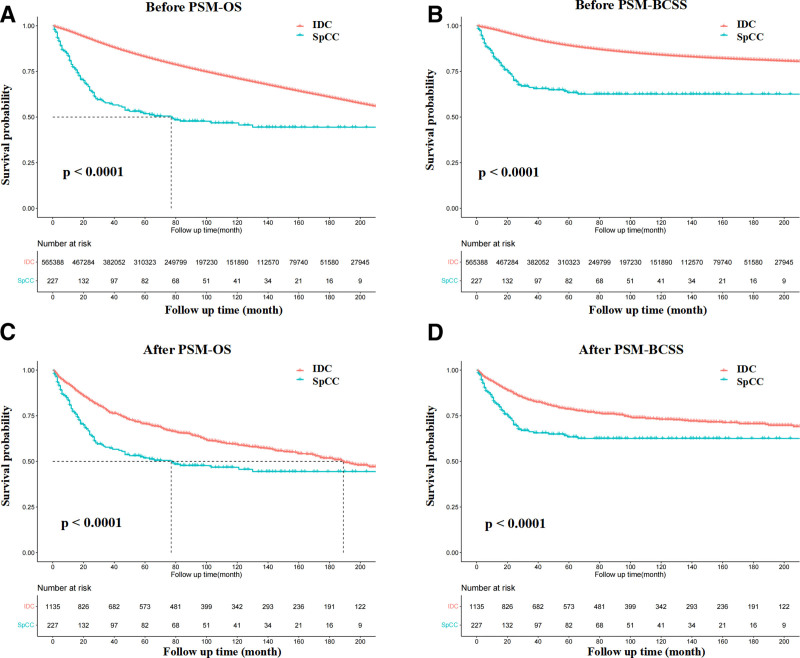
Comparisons of survival between SpCC and IDC of the breast. (A and B) OS and BCSS of IDC and SpCC cohorts before PSM. (C and D) OS and BCSS of IDC and SpCC cohorts after PSM. BCSS = breast cancer cause-specific survival, IDC = invasive ductal carcinoma, OS = overall survival, PSM = propensity score matching, SpCC = spindle cell carcinoma.

**Figure 4.**
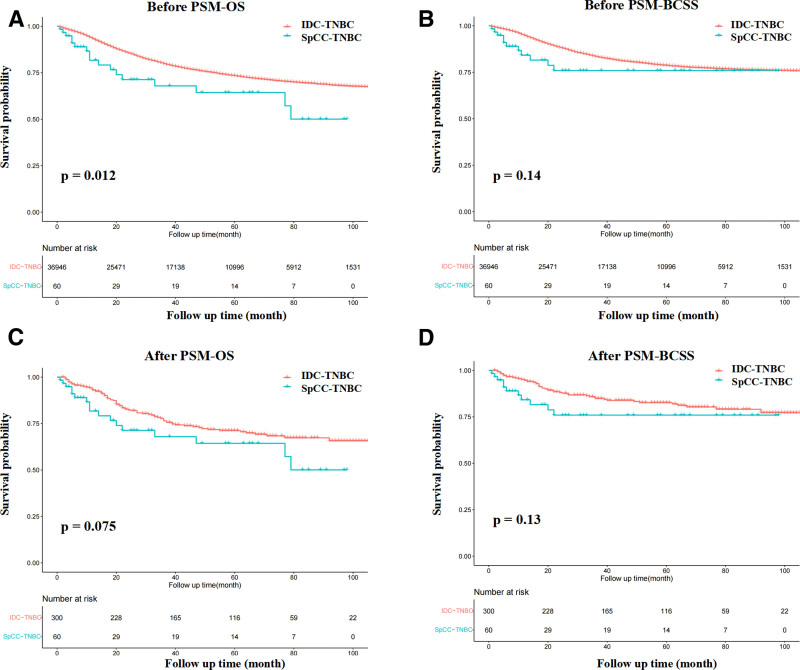
Comparisons of survival between IDC-TNBC and SpCC-TNBC cohorts. (A and B) OS and BCSS of IDC-TNBC and SpCC-TNBC cohorts before PSM. (C and D) OS and BCSS of IDC-TNBC and SpCC-TNBC cohorts after PSM. BCSS = breast cancer cause-specific survival, IDC = invasive ductal carcinoma, OS = overall survival, PSM = propensity score matching, SpCC = spindle cell carcinoma, TNBC = triple-negative breast cancer.

Based on the Kaplan–Meier plot, SpCC showed a significantly worse outcome than breast IDC (Fig. 3, both *P* < .0001). The 5-year OS rates in SpCC and IDC were 59% and 86%, respectively, and the 5-year BCSS rates in SpCC and IDC were 70%, and 91%, respectively. We further compared BCSS and OS between the IDC-TNBC and SpCC-TNBC subgroups. The results are presented in Figure 4. The OS of the SpCC-TNBC subgroup was worse than that of the IDC-TNBC subgroup (*P* = .012). However, no statistical significance was observed in the BCSS between the IDC-TNBC and SpCC-TNBC cohorts.

To eliminate the uneven distributions in baseline characteristics, we performed a 1:5 (SpCC/IDC) PSM analysis to investigate the survival outcomes between the 2 groups. Finally, 1135 IDC patients were selected to match 227 SpCC patients. No significant differences were observed for any of the baseline variations between the matched groups (Table 1). The patients with SpCC exhibited a poorer clinical outcome than IDC patients (Fig. 3, both *P* < .0001). The 5-year OS rates in SpCC and IDC were 59% and 75%, respectively, and the 5-year BCSS rates in SpCC and IDC were 70%, and 82%, respectively.

We also used the same strategy to match SpCC-TNBC. We obtained 60 patients with SpCC-TNBC and 300 matched patients with IDC-TNBC. There were no significant differences in the baseline variations between the 2 groups (Table 2). However, no statistical significance was observed in the OS and BCSS between patients from the IDC-TNBC and SpCC-TNBC cohorts (Fig. 4).

**Figure 5.**
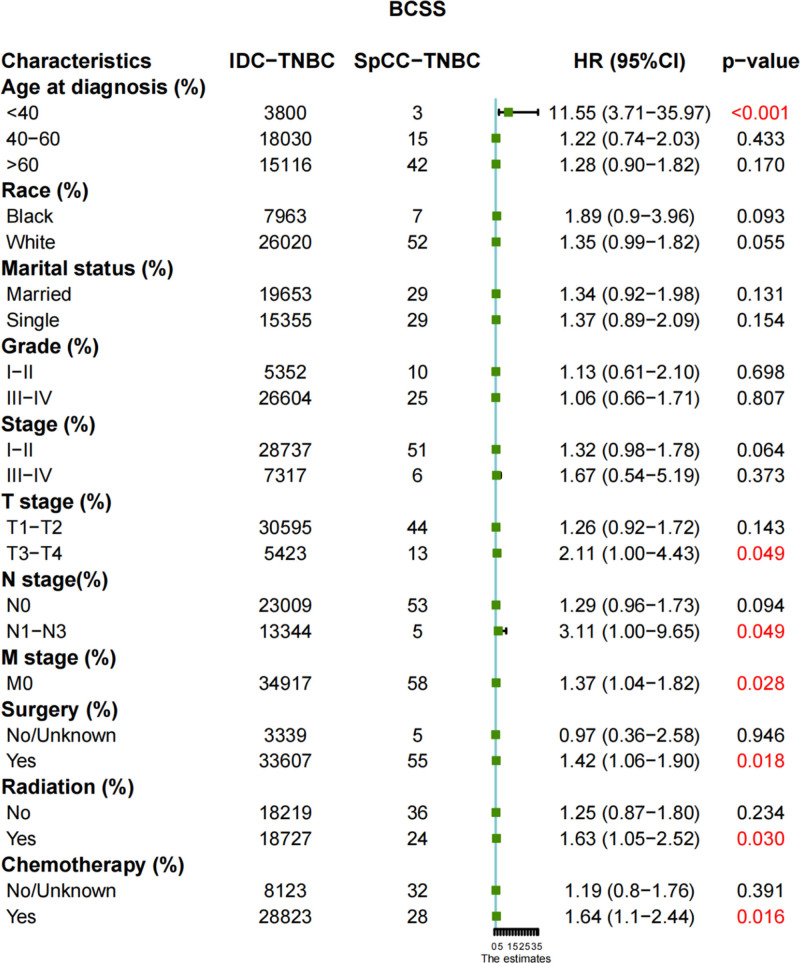
Subgroup analysis of BCSS between IDC-TNBC and SpCC-TNBC cohorts. BCSS = breast cancer cause-specific survival, IDC = invasive ductal carcinoma, SpCC = spindle cell carcinoma, TNBC = triple-negative breast cancer. Unknown: The receptor status is unknown, possibly due to undetected, test failure, or the result not being recorded.

We found that there was no difference in BCSS between the IDC-TNBC and SpCC-TNBC groups before or after PSM. Therefore, we further conducted subgroup analysis of BCSS. As shown in Figure 5, the patients with SpCC-TNBC had worse BCSS than IDC-TNBC in the subgroups of age <40 years old, T3-T4 stage, N1 to N3 stage, receiving chemotherapy, receiving radiotherapy and receiving surgery.

**Figure 6.**
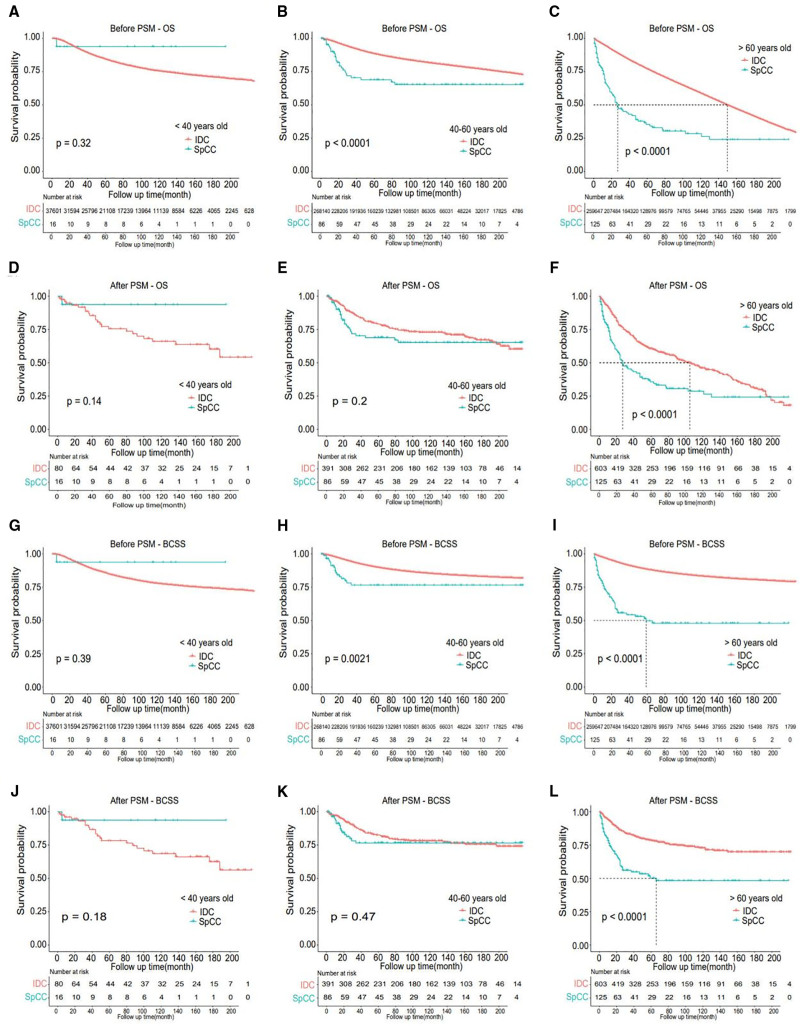
Age subgroup analysis of OS and BCSS between IDC and SpCC cohorts. (A–C) OS of IDC and SpCC cohorts in age subgroup (age < 40 yr old, 40–60 yr old and > 60 yr old) before PSM. (D–F) OS of IDC and SpCC cohorts in age subgroup (age < 40 yr old, 40–60 yr old and > 60 yr old) after PSM. (G–I) BCSS of IDC and SpCC cohorts in age subgroup (age < 40 yr old, 40–60 yr old and > 60 yr old) before PSM. (J–L) BCSS of IDC and SpCC cohorts in age subgroup (age < 40 yr old, 40–60 yr old and > 60 yr old) after PSM. BCSS = breast cancer cause-specific survival, IDC = invasive ductal carcinoma, OS = overall survival, PSM = propensity score matching, SpCC = spindle cell carcinoma.

The proportion of patients older than 60 years of age with SpCC was higher than that of IDC, and the patients with SpCC had worse OS and BCSS than IDC among these older patients before or after PSM (Fig. 6). But there were no significant differences in OS or BCSS in the populations younger than 40 or 40 to 60 years old (Fig. 6).

### 3.3. Identifying prognostic factors for SpCC

We also explored the potential prognostic factors in breast SpCC using multivariate Cox regression analysis. As shown in Table 3, age at diagnosis, T stage, N stage, distant metastasis and no or unknown surgery were all significantly associated with poor BCSS and OS. HER2 status was also a prognostic factor for OS, and PR status was also a prognostic factor for BCSS. We did not find that race, marital status, tumor grade, ER status, radiotherapy and chemotherapy were associated with OS or BCSS.

**Table 3:** Prognostic factors for breast cancer caused-specific survival (BCSS) and overall survival (OS) in breast SpCC by multivariate Cox regression analysis.

Characteristics	OS			BCSS		
	HR	95% CI	*P*-value	HR	95% CI	*P*-value
Age at diagnosis						
<40						
40–60	4.60	0.61–34.73	.139	2.74	0.35–21.29	.337
>60	24.33	3.28–180.57	.002	17.12	2.29–128.09	.006
Race						
Black						
White	1.14	0.53–2.46	.741	1.03	0.42–2.52	.941
Other	0.82	0.16–4.24	.812	1.09	0.19–6.37	.926
Marital status						
Married						
Single	1.18	0.75–1.84	.481	1.03	0.58–1.80	.928
Unknown	1.51	0.58–3.93	.394	1.13	0.35–3.62	.841
Laterality						
Left						
Right	1.06	0.68–1.65	.814	0.72	0.41–1.27	.262
Grade						
I–II						
III–IV	1.59	0.83–3.05	.166	2.25	0.85–6.00	.104
Unknown	1.57	0.77–3.21	.216	2.01	0.70–5.78	.194
Stage						
I–II						
III–IV	0.51	0.17–1.52	.230	0.49	0.14–1.73	.268
Unknown	3.39	0.37–30.81	.277	15.61	1.16–210.81	.039
T stage						
T1–T2						
T3–T4	5.04	2.7–9.42	<.001	7.55	3.50–16.31	<.001
Unknown	1.88	0.39–8.98	.430	2.78	0.44–17.47	.276
N stage						
N0						
N1–N3	3.75	1.42–9.88	.008	5.70	1.88–17.30	.002
Unknown	0.24	0.03–1.65	.147	0.11	0.01–1.18	.068
M stage (%)						
M0						
M1	6.38	1.57–25.94	.010	6.00	1.31–27.46	.021
Unknown	3.76	0.71–19.82	.118	1.36	0.26–7.25	.717
ER status						
Negative						
Positive	0.92	0.32–2.63	.872	0.33	0.08–1.42	.137
Borderline/Unknown	0.56	0.07–4.27	.573	2.81	0.03–249.64	.651
PR status						
Negative						
Positive	1.88	0.65–5.43	.245	7.23	1.71–30.58	.007
Borderline/Unknown	0.70	0.10–4.78	.715	0.20	0–16.39	.471
HER2 status						
Negative						
Positive	11.35	1.09–118.33	.042	4.50	0.36–56.79	.245
Borderline/Unknown	0.56	0.03–9.03	.681	1.23	0.07–20.42	.883
Subtypes						
HR−/HER2−						
HR+/HER2−	0.48	0.13–1.70	.256	0.26	0.05–1.24	.091
Unknown	2.70	0.16–46.97	.495	1.12	0.06–20.87	.938
Surgery						
No/Unknown						
Yes	0.23	0.10–0.54	.001	0.24	0.09–0.66	.006
Radiation						
No						
Yes	0.91	0.56–1.49	.705	1.03	0.56–1.90	.919
Chemotherapy						
No/Unknown						
Yes	1.03	0.62–1.71	.897	1.11	0.60–2.05	.736

Borderline: The test result falls within a borderline range and cannot be definitively classified as positive or negative. Unknown: The receptor status is unknown, possibly due to undetected, test failure, or the result not being recorded.

BCSS = breast cancer cause-specific survival, OS = overall survival, SpCC = spindle cell carcinoma.

## 4. Discussion

SpCC of the breast is a rare subtype of invasive breast malignancy. Previous studies on the clinical features of SpCC of the breast have been greatly restricted by case reports and small case series.^[15–17]^ In the present study, we discussed the differences in clinicopathological features, patterns of treatment and survival outcomes of SpCC compared to IDC of the breast in a large cohort derived from the SEER database for the first time, which could provide a persuasive reference for clinical practice.

In the present study, we found that SpCC of the breast had distinct clinicopathological characteristics. Compared with IDC, SpCC presented with higher cT stage, lower rate of nodal metastasis, higher rate of distant metastasis and higher proportion of TNBC. These findings were similar to those of previous studies.^[18,19]^ SpCC has the features of a soft tissue sarcoma. The tumor often grows rapidly, whereas the frequency of lymph node metastasis is low in SpCC.^[19]^ Previous studies reported that the axillary node metastasis rate of SpCC ranged from approximately 0 to 26%, which is lower than that of common breast cancer.^[20–24]^ Therefore, it would be rational to perform a sentinel lymph node biopsy to avoid unnecessary axillary dissection in the treatment plan of SpCC. In addition, SpCC showed a worse prognosis than IDC. To our knowledge, this is the first and largest study to compare the clinical outcome of SpCC with IDC of the breast.

We also performed PSM analysis to eliminate the potential influence of prognostic confounders on the accuracy of the results. A significant difference in prognosis was observed. To our knowledge, this is the first study to apply PSM to the analysis of SpCC of the breast.

Considering the large proportion of the TNBC subtype in the SpCC cohort, we performed a separate analysis and investigated the characteristics in this subgroup. While aggressive disease features of TNBC subgroup patients were not revealed compared to those with IDC, the OS of the SpCC-TNBC subgroup was worse than that of the IDC-TNBC subgroup. However, no significant difference was found in the BCSS between the 2 groups. Unfortunately, the small number of cases makes it difficult to draw a convincing conclusion, and more data are needed to explore the potential causes.

In further subgroup comparisons, the patients with SpCC-TNBC had worse BCSS than IDC-TNBC in the subgroups of younger age, larger tumor size, later lymph node stage, chemotherapy, radiotherapy and surgery. In addition, the patients with SpCC had worse OS and BCSS than IDC in the subgroup of older than 60 years in the subgroup analysis based on age. However, few studies have focused on the molecular mechanism of SpCC-TNBC. A recent study revealed that the spindle cell component tends to have higher programmed death ligand 1 (PD-L1) expression than the epithelial component.^[15]^ Since TNBC has the highest level of PD-L1 expression, PD-L1 could be a target for immunotherapy.^[25,26]^ Several KEYNOTE series findings have demonstrated that PD-L1 inhibitors in combination with chemotherapy can produce optimal outcomes in the treatment of TNBC.^[26]^ We aim to explore potential therapeutic effects of immune checkpoint inhibitors in patients with SpCC-TNBC in future work. For those with younger age or older age and later stages, special attention should be given to standardized full-course treatment, and regular follow-up in the future is also very important. Moreover, further studies are urgently needed to explore the underlying molecular features of SpCC-TNBC.

We also explored the potential prognostic factors in SpCC of the breast through multivariate Cox regression analysis. We found that older age, advanced T stage, N stage or M stage were all risk factors for SpCC, which were similar to the results reported by prior studies on other histologic types of breast cancer.^[27,28]^ These findings further substantiate the need for standardized treatment protocols and more rigorous follow-up strategies for SpCC patients with older age or later stages. Studies indicated that the absence of PR reflected a nonfunctional ER pathway.^[29]^ Previous studies also showed that ER(+)/PR(+) breast cancers can respond better to hormone therapy than ER(+)/PR(−) breast cancer and have better BCSS.^[30–32]^ Strangely, we found that PR positive was a risk factor for BCSS, while ER was not associated with either OS or BCSS in SpCC of the breast. A possible reason for this finding is that the majority of cases in SpCC are TNBC, resulting in a relatively small number of ER(+)/PR(−) cases. This imbalance in the cohort may lead to the counterintuitive conclusion. In addition, it may be related to the detection method and spindle cell tumors of the breast may demonstrate some hormone receptor positive staining from surrounding breast tissue on cytology.^[19]^ We also found that HER2 positive was a risk factor for OS. HER2 status was only available from January 2010 and the number of HER2-positive cohort was quite small. The small HER2-positive cohort warrants further investigation in larger patient populations to validate this result. With the expansion of the SEER database in the future, more comprehensive and accurate information on prognostic factors for SpCC of the breast will be revealed.

Our study also had several limitations. First, there is a proportion of missing data among some important clinical variables, including HER2 status available from January 2010. The SEER database lacks information on chemotherapy regimens, cycles or dosages. These critical missing data might result in underestimation or misestimation and potentially weaken the power of our results. Second, only patients with comprehensive clinical features were enrolled in our retrospective study. Hence, there may be selection bias in this study. Last, some details of systemic therapy were unavailable in the SEER database, such as hormonal therapy and targeted therapy. Thus, the associations between these systemic therapies and the outcomes need to be further clarified in future studies. With the expansion of the SEER database in the future, comprehensive information for SpCC of the breast will be available. Although limitations existed, our study indeed improved our understanding of this rare entity.

## 5. Conclusion

In summary, our study revealed that SpCC of the breast had individual clinicopathological characteristics. SpCC of the breast presented with increasing aggressive behavior in comparison with IDC and worse clinical outcomes than IDC for both the whole group and the TNBC subgroup. We should perform sentinel lymph node biopsy in the treatment of clinically node-negative SpCC patients. Standardized full-course treatment, and regular and close follow-up in the future is also very important for SpCC. Patients with SpCC should be considered candidates for innovative therapeutic regimens. Future studies will explore the molecular features of SpCC-TNBC and the potential therapeutic efficacy of immunotherapy against this disease.

## Acknowledgments

The authors sincerely thank the Surveillance, Epidemiology, and End Results (SEER) program for their efforts in establishing the SEER database.

## Author contributions

**Conceptualization:** Yushi Sun, Yang Liu.

**Data curation:** Yushi Sun.

**Formal analysis:** Yushi Sun, Heyan Chen.

**Funding acquisition:** Yang Liu.

**Methodology:** Yushi Sun, Heyan Chen, Ke Wang.

**Supervision:** Ke Wang, Yang Liu.

**Writing – original draft:** Yushi Sun.

**Writing – review & editing:** Ke Wang.
